# Supplementary material for: Nociplastic Pain in Multiple Sclerosis Spasticity: Dermatomal Evaluation, Treatment with Intradermal Saline Injection and Outcomes Assessed by 3D Gait Analysis: Review and a Case Report
Source: Int J Environ Res Public Health. 2022 Jun 27;19(13):7872. doi: 10.3390/ijerph19137872 (PMC9266269; doi:10.3390/ijerph19137872)
Supplement: Supplementary file 1 [file ijerph-19-07872-s001.zip › ijerph-1749860-supplementary.pdf]

.

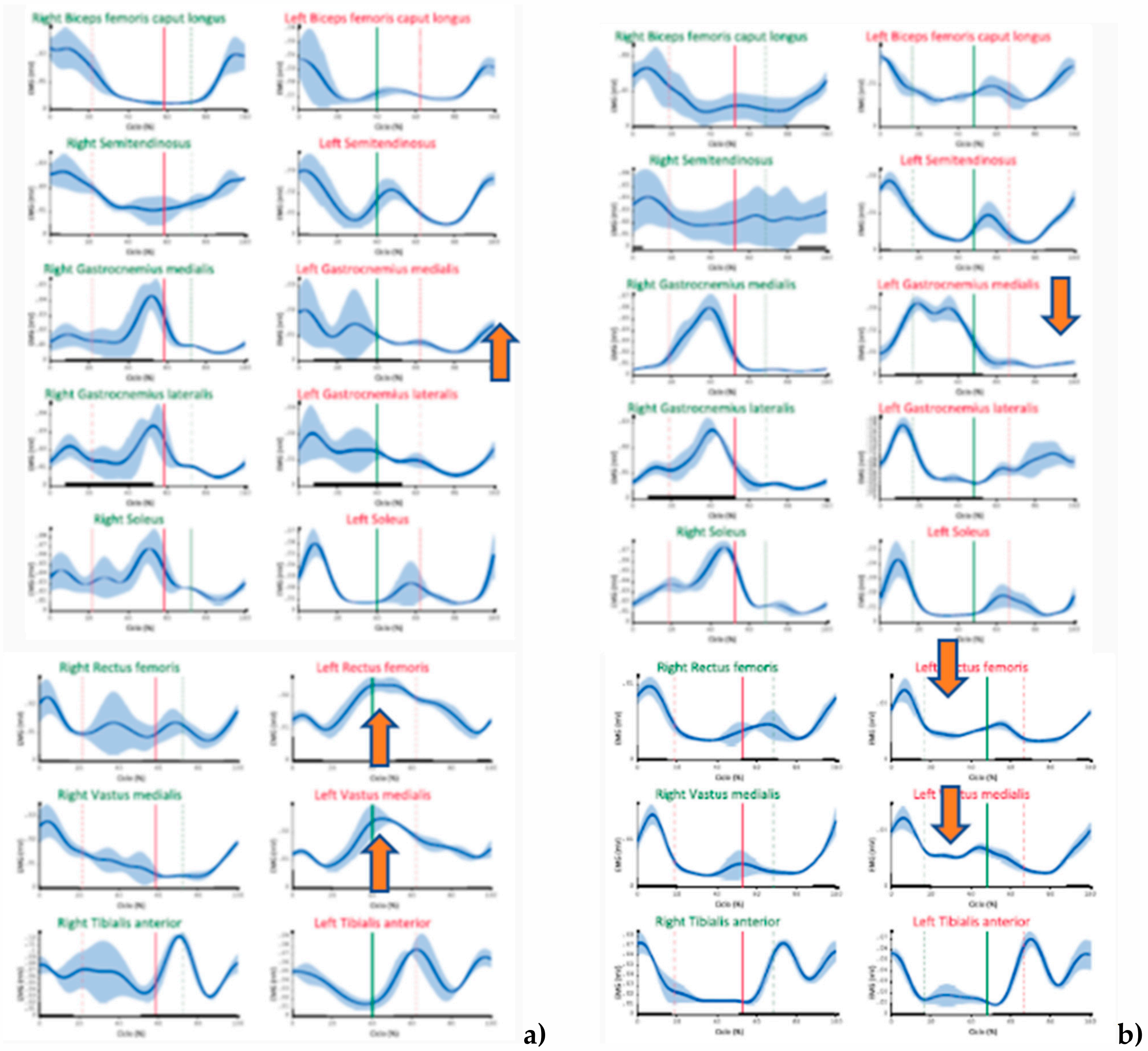


T 0 T 1

**Supplementary files:**

**Figure S1. Surface-EMG signals analysis are shown about muscle activation timing before treatment at T0 (a) and five minutes after treatment at T1 (b).**

Orange arrows indicate muscle hyperactivity of the rectus femoris, vastus medialis and gastrocnemius medialis out of normal phase at T0 and mostly in physiological phase at T1
